# Peer support for people living with rare or young onset dementia: An integrative review

**DOI:** 10.1177/14713012221126368

**Published:** 2022-09-16

**Authors:** Mary Pat Sullivan, Veronika Williams, Adetola Grillo, Roberta McKee-Jackson, Paul M Camic, Gill Windle, Joshua Stott, Emily Brotherhood, Sebastian J Crutch, on behalf of the Rare Dementia Support (RDS) research team

**Affiliations:** Faculty of Education and Professional Studies, 6057 Nipissing University, North Bay, ON, Canada; UCL Institute of Neurology, Dementia Research Centre, 58673 University College London, London, UK; Ageing and Dementia @ Bangor, Dementia Services Development Centre (DSDC), School of Health Sciences, 1506 Bangor University, Bangor, UK; Department of Clinical, Educational and Health Psychology, 58673 University College London, London, UK; UCL Institute of Neurology, Dementia Research Centre, 58673 University College London, London, UK; UCL Institute of Neurology, Dementia Research Centre, 58673 University College London, London, UK

**Keywords:** rare dementia, young onset dementia, peer support, integrative review, relationality

## Abstract

**Objectives:**

The aim of this integrative review was to identify and synthesize the literature on peer support interventions for people living with or caring for someone with a rare or young onset dementia.

**Design:**

A literature search of articles was performed using the Nipissing University Primo search system, a central index that enables simultaneous searches across databases which included MEDLINE (PubMed), Web of Science, PsycINFO, CINAHL, Sociological Abstracts, Cochrane Library.

**Results:**

The eleven papers that met the inclusion criteria spanned eighteen years and from five countries. Studies reported on peer support programs that were either hospital-based (n = 6) or community-based (n = 4), and were predominantly led by disciplines in the health sciences. Only one study did not involve delivering services. There was a range of methodological quality within the studies included in the review. Further analysis and synthesis led to the identification of three overarching peer support themes. These included: (1) peers as necessarily part of social support interventions; (2) a theoretical portmanteau; and (3) dementia spaces and relationality.

**Conclusion:**

Consistent with a much larger body of work examining peer involvement in social interventions, this review reinforced the valuable contribution of peers. A full understanding of the mechanisms of change was not achieved. Notwithstanding, the issue of studies neglecting to sufficiently conceptualize and describe interventions is an important one – drawing attention to the need to continue to explore varied delivery, including co-produced models, and more effective evaluation strategies to inform the dementia care sector.

## Introduction

The World Alzheimer Report (Gauthier et al., 2021) estimates that there are 55 million people living with dementia worldwide. The report also indicates that only 25% have a diagnosis and 30% are misdiagnosed. Within these figures are those living with a rare, inherited, or young onset dementia (see, for example, Murray et al., 2011; Tang-Wai et al, 2004). While the primary cause of dementia is Alzheimer’s disease (AD), Harvey et al. (2003) suggest that atypical or vascular causes may account for approximately 25% of all diagnoses. Atypical forms of dementia are more likely to be diagnosed in individuals under the age of 65 (Brotherhood et al., 2019). Recently, Hendricks et al. (2021) calculated that there are 3.9 million people between the ages of 30 – 60 living with young onset dementia.

Individuals who are diagnosed with dementia at a younger age face a myriad of intersecting bio-medical, life stage and structural challenges that are increasingly recognized within the literature. Foremost, age, atypical symptom profiles and a lack of specialist neurological services for those living outside large urban centres often result in a delayed or inaccurate diagnosis (Canadian Academy of Health Sciences, 2019). Individuals not only face a future of neurodegenerative decline, but the loss of employment, unexpected marital and childcare transitions, disrupted relationships, and social exclusion due to numerous systemic barriers preventing full citizenship participation (Mayrhofer et al., 2018; Millenaar et al., 2016; Sonnicksen, 2016). Problem-solving and coping due to these psychosocial circumstances are also hindered by an absence of dementia services that can flexibility tailor supports for individuals with a non-Alzheimer’s diagnosis, who are younger in age and who may require a family-centred approach to care (Harris & Keady, 2009; Novek & Menec, 2021).

Peer support, while long familiar in the mental health and disability sectors, has recently achieved more prominence within dementia care. Peer support has been defined in a variety of ways but is generally presented as “a system of giving and receiving help founded on key principles of respect, shared responsibility, and mutual agreement of what is helpful” (Mead et al., 2001: p. 135). Positively received by service users and providers within the health and social care sectors, virtual or face-to-face peer support for care partners as a supplement to professional support are commonplace. Peer support for people living with dementia is less widespread, although opportunities for socialization among peers is common in many not-for-profit dementia organizations. For individuals affected by a rare or young onset dementia, tailored peer support or opportunities to engage with peers is patchy at best (Brotherhood et al., 2020). This gap in support means that their access to peers is by connecting with others who are associated with multiple different conditions, dementia stages and ages.

There is a growing body of literature on peer support in dementia care reporting a variety of positive outcomes for people living with dementia and care partners. A recent scoping review on peer support (Carter et al., 2020) and a systematic review on support interventions for care partners (Dam et al., 2016), however, have identified various methodological limitations in this body of literature. Therefore, there are still gaps in the evidence of what works to facilitate an adoption of peer support best practice models in service delivery. Nevertheless, outcomes for care partners are reported to include improvements in understanding dementia and care strategies through experiential sharing, a sense of belonging, feeling less alone, reduction in stress and anxiety, and sharing and empathy (e.g., Lauritzen et al., 2015; Smith et al., 2018; Willis et al., 2016). Although understandings are more restricted for people living with dementia, outcomes include reduced loneliness and isolation, and improved overall wellbeing (e.g., Theurer et al., 2015; Willis et al., 2016). The homogenization of peer support delivery means it is difficult to generalize from these studies as to whether either the models and/or the outcomes are meaningful specifically for people living with a rare or young onset dementia and their care partners.

## Methods

### Aims

The aim of this integrative review was to identify and synthesize the literature on peer support interventions for people living with or caring for someone living with rare or young onset dementia. Research questions were:

1. What are the characteristics of people living with rare or young onset dementia and/or their care partners investigated in the literature?
2. How is peer support conceptualized in the literature? What are the theories or mechanisms of change in peer support?
3. What are the specific interventions (or components of interventions) using peer support (e.g., supportive counselling, telephone support, education, social/recreational), how is it delivered, and what are the reported outcomes?
4. What is the methodological quality of the available evidence on peer support in rare or young onset dementia care and support?

The review protocol was registered with PROSPERO, an international prospective register of systematic reviews (ID CRD42020164951).

### Design

Given design heterogeneity among the studies within the literature an integrative review was adopted. Looking both broadly and critically at the area of interest, an integrative review includes: (1) problem identification; (2) systematic literature search; (3) data quality appraisal; (4) analysis and synthesis; and (5) presentation and dissemination (Toronto, 2020). The Preferred Reporting Items for Systematic Reviews and Meta-Analyses (PRISMA) was followed to report this review (Page et al., 2021). The quality appraisal was conducted using the Joanna Briggs Institute (JBI) appraisal tools for qualitative research and quasi-experimental (non-randomized) studies (JBI, 2020) and the Mixed Methods Appraisal Tool (MMAT) (Hong et al., 2018). Data synthesis was consistent with an integrative convergent design (Noyes et al., 2019).

### Search strategy

A literature search was performed in February 2021 and updated in December 2021 using the Nipissing University Primo search system, a central index that enables simultaneous searches across databases to which the library is subscribed as well as content beyond the university’s collection. The databases included MEDLINE (PubMed), Web of Science, PsycINFO, CINAHL, Sociological Abstracts, Cochrane Library using different iterations of the following search terms: ‘peer support’; ‘peer mentoring’; ‘peer befriending’; ‘peer volunteering’; ‘dementia’; ‘young onset dementia’; ‘early onset dementia’; ‘young onset alzheimer’s disease’; ‘early onset alzheimer’s disease’; ‘frontotemporal dementia (FTD)’; ‘familial FTD’; ‘dementia with Lewy bodies’; ‘posterior cortical atrophy’; ‘familial Alzheimer’s disease’; ‘primary progressive aphasia (PPA)’, and Boolean operators, ‘AND’ and ‘OR’. Additional articles were obtained by searching reference lists of included studies. Less common dementias may be categorized using different terms (e.g., young onset, early onset, rare dementia). ‘Rare dementia’ was not included as a search term because it provided very few results in a pilot search. The specific diseases or conditions included as search terms were those that are more common among rarer forms.

### Inclusion/exclusion criteria

The population of interest in this review included persons 18 years and older, living with or caring (e.g., spouse/partner, child, other relative or friend) for someone living with a rare or young onset dementia, defined as people living with a rare dementia at any age or people living with dementia with a younger age of onset (that is, under 65 years). Diseases or conditions of interest included including Alzheimer’s disease, FTD, Lewy body dementia or other less common forms (e.g., primary PPA, posterior cortical atrophy, familial FTD, familial Alzheimer’s disease). Peer support included any type of program delivered alone or with other interventions (i.e., multicomponent) that involved peers who possess experiential knowledge of living with or caring for someone living with dementia or a part of natural or embedded social networks (such as family, friends, or neighbours) (Dennis, 2003).

Studies were included if they focused on the population of interest and the program as described above, published in English language, and were primary research studies using any methodology (qualitative, quantitative, or mixed methods). There was no time limitation to the publication date. Studies were excluded if the population of interest was older than 65 years and the type of dementia was not reported, support interventions included peers and non-peers or paid peers and did not report primary data.

### Study selection and data extraction

**Figure 1.**
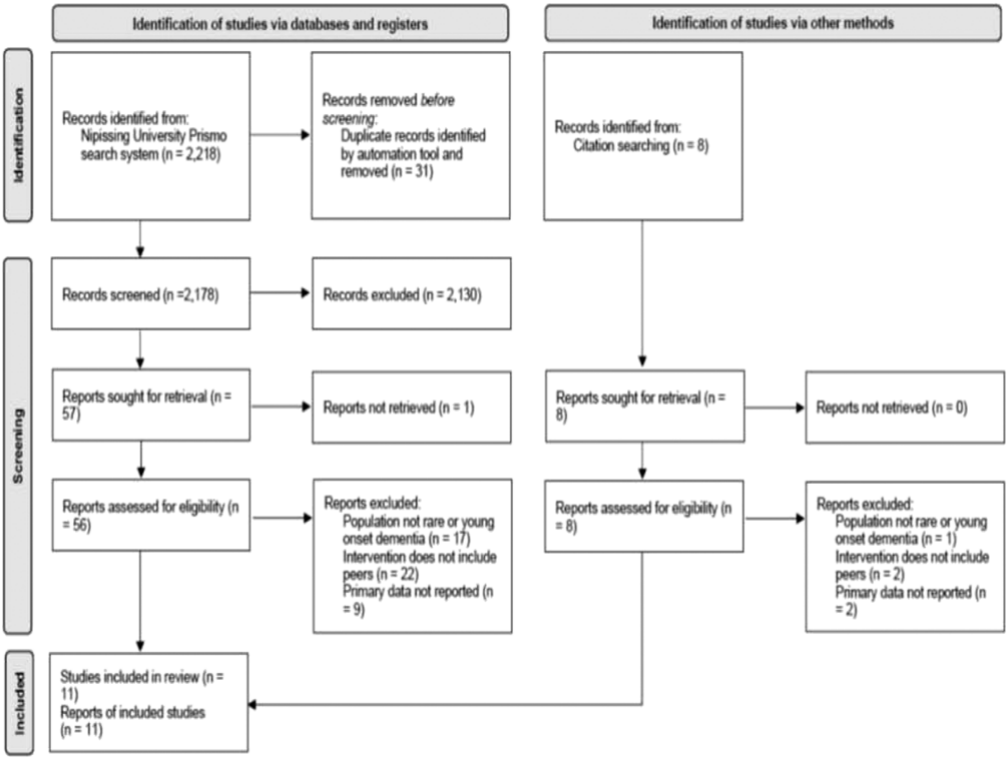
PRISMA flow diagram showing the process of selecting studies for the review.

The selection process for the review is represented in Figure 1. Records retrieved from the Primo search were imported into Rayyan, a free web and mobile app that provides semi-automation for screening articles (Ouzzani et al., 2016). Duplicates were identified by Rayyan and removed. One reviewer (AG) screened titles and abstracts after duplicates were removed. Full texts of 56 papers considered eligible for review were screened independently by two reviewers (AG, MPS) and discrepancies were resolved through discussion between both reviewers. Forty-eight papers were excluded because they did not meet the inclusion criteria. An additional nine papers were obtained by searching reference lists of included papers. The two reviewers independently screened the full texts of these papers resulting in the exclusion of five papers that did not meet the inclusion criteria. A total of 11 studies were included in this review.

No authors were contacted for further information. A bespoke data extraction tool was created and included information on study characteristics including year of publication, country of origin, aims, design, sample size and methodology. Data extraction was undertaken by the two reviewers.

### Quality assessment

The quality of included papers was evaluated by three reviewers (AG, MPS, VW) independently using the JBI Checklist Tools (2020) and in one instance the MMAT (Hong et al., 2018), with disagreements resolved by a second evaluation and further discussion until consensus was reached. No papers were excluded despite some being considered of low overall quality.

### Data analysis and synthesis

Data analysis and synthesis was completed by two reviewers (MPS and VW). Both data immersion and reduction were completed by the creation of an enhanced data matrix which focused and organized the data (i.e., objective of intervention, conceptual background, delivery, outcomes) and memoing throughout this process (Toronto & Remington, 2020). Qualitizing the data occurred at this time whereby descriptive statistics in results sections were assigned words and/or phrases (Noyes et al., 2019). This was followed by an inductive coding process assisted by Atlas.ti version 8 to facilitate the development of themes relevant to the research questions (Braun & Clarke, 2006).

## Results

### Study Characteristics

Eleven papers included in the review spanned 18 years and from a range of countries including: Canada (n = 4), United Kingdom (n = 4), Australia (n = 1), US (n = 1) and Germany (n = 1). Studies reported on support programs that were either hospital-based (n = 6) (Diehl et al., 2003; Jokel et al., 2017; Marziali & Climans, 2009; Morhardt et al., 2019; O’Connell et al., 2014; Taylor-Rubin et al., 2020) or community-based (n = 4) (Carone et al, 2016; Clare et al., 2008; Davies-Quarrell et al., 2010; Phinney et al., 2016) and were predominantly led by disciplines in the health sciences. Only one study did not involve delivering services (Stamou et al., 2020).

Four papers were published between 2003 and 2010 describing interventions for frontotemporal dementia (FTD) (n = 2) (Diehl et al., 2003; Marziali & Climans, 2009), a support program established by people living with dementia (n = 1) (Davis-Quarrell, 2010) and an internet-based self-help network (n = 1) (Clare et al., 2008). The remaining seven papers published between 2016 and 2020 were interventions for primary progressive aphasia (PPA) (n = 3) (Jokel et al., 2017; Morhardt et al., 2019; Taylor-Rubin et al., 2020), social/recreational programs (n = 2) (Carone et al., 2016; Phinney et al., 2016), video-conferencing support group for people in rural settings (n = 1) (O’Connell et al., 2014) and exploring post-diagnostic needs of people living with or caring for someone living with dementia (n = 1) (Stamou et al., 2020).

Across most studies the sample sizes were small. Three studies had a sample size of ≤10 (Clare et al., 2008; Diehl et al., 2003; O’Connell et al., 2014). Five studies had a sample size range of 12–25 (Carone et al., 2016; Jokel et al., 2017; Marziali & Climans, 2009; Morhardt et al., 2019; Phinney et al., 2016). One study had 38 participants (Taylor-Rubin et al., 2020), another 233 participants (Stamou et al., 2020) while the other did not report on sample size (Davis-Quarrell, 2010). Two studies reported mixed education and ethnocultural characteristics among participants (Jokel et al., 2017; Stamou et al., 2020), one study reported all participants had a similar ethnocultural background (Carone et al., 2016) and another study reported participants having a similar education level (Diehl et al., 2003). Half of the interventions (n = 5) were designed for both the person living with dementia and their care partner (Carone et al., 2016; Davies-Quarrell, 2010; Jokel et al., 2017; Morhardt et al., 2019; Taylor-Rubin et al., 2020) while the remaining were either solely for the person living with dementia (n = 2) (Clare et al., 2008; Phinney et al., 2016) or care partner (n = 3) (Diehl et al., 2003; Marziali & Climans, 2009; O’Connell et al., 2014). Most care partners were spouses/partners and female. Five studies focused on a specific diagnosis (PPA or FTD) (Diehl et al., 2003; Jokel et al., 2017; Marziali & Climans, 2009; Morhardt et al., 2019; Taylor-Rubin et al., 2020), two studies had mixed diagnoses (O’Connell et al., 2014; Stamou et al., 2020) while the remaining four did not specify type of dementia (Carone et al., 2016; Clare et al., 2008; Davies-Quarrell et al., 2010; Phinney et al., 2016). The paper reporting on post-diagnostic support needs included people living with dementia and care partners (Stamou et al., 2020). A summary of the included studies is provided in Table 1.

**Table 1.** Summary of included studies (n = 11).

Author, date, country	Aim	Design	Sample	Data collection	Data analysis
Carone, et al. (2016) United Kingdom	To explore the needs of men living with early onset dementia and the impact of a community-based sports group.	Qualitative	PLWD^a^ (n = 5), spousal care partner (n = 5), coaching staff (n = 5), group facilitator (n = 5)	Individual or paired interviews, staff and family focus groups.	Thematic analysis (Braun & Clarke, 2006) and follow-up member checking.
Clare et al. (2008) United Kingdom	To investigate the experience belonging to an international internet-based self-help network for people living with dementia and its impact on self-concept and adjustment; and factors that promote self-help, mutual support and advocacy.	Qualitative longitudinal	PLWD (n = 7)	Semi-structured interviews conducted by email.	Interpretative phenomenological analysis (Smith et al., 1999)
Davies-Quarrell et al. (2010) Wales	To evaluate a relationship-centred club for people with young onset dementia.	Qualitative “self-evaluation” (p. 45)	Not reported	Not reported	Not reported
Diehl et al. (2003) Germany	To evaluate an out-patient support group and self-help meetings for care partners of people living with FTD.	Qualitative	Spousal care partner (n = 8)	Post-intervention evaluation at two time points – group interview immediately after intervention using an adapted questionnaire for evaluating AD support groups (Yale, 1995) and author developed 14-item questionnaire mailed 6-months after the group.	Not reported
Jokel et al. (2017) Canada	To assess feasibility and effectiveness of an outpatient group intervention for people living with PPA and their spouses.	Quasi-experimental comparison group	Spousal dyad (n = 5 per group)	Pre- and post-administration of 17-item ASHA quality of communication life Scale (Paul et al., 2004) and author developed spousal questionnaire, video-recorded communication strategies, evaluation forms and email messages.	Nonparametric statistics (Wilcoxon signed-rank test), ratings of video-recorded communication strategies and qualitative analysis of feedback from evaluation forms and email messages.
Marziali & Climans (2009) Canada	To evaluate the outcomes a videoconferencing education and support group for spousal care partners of people with FTD and the use of technology to access a health service program.	Qualitative	Spousal care partner (n = 12)	Interviews	Not reported
Morhardt et al. (2019) USA	To evaluate the development and feasibility of a Memory Clinic’s psycho-educational support group for people living with PPA and their care partners.	Qualitative	PLWD (n = 9) Care partner (n = 8)	Observational field notes, group transcriptions	Thematic analysis
O'Connell et al. (2014) Canada	To evaluate a videoconferencing support group for rural dwelling spousal care partners offered by a specialist Memory clinic.	Qualitative	Spousal care partner (n = 10)	Transcribed in-person group discussions held 18 months after the group began, attendance records, and field notes of facilitators.	Thematic analysis (Braun & Clarke, 2006)
Phinney et al. (2016) Canada	To explore how a community-based club promotes social citizenship for people with young onset dementia.	Qualitative	PLWD (n = 12–15) Family care partners (n = 30)	Ethnographic approach including participant observation of group walks, “go-along” interviews, focus group discussions (using photo elicitation), field notes and anonymous family satisfaction survey.	Inductive analysis (Thomas, 2006)
Stamou et al. (2020) United Kingdom	To explore positive experiences of post diagnostic support services for people with young onset dementia.	Qualitative survey	PLWD (n = 48), care partner (n = 101), dyad (n = 84)	Cross-sectional semi-structured survey	Descriptive statistics, thematic analysis
Taylor-Rubin et al., 2020 Australia	To evaluate an outpatient PPA-specific education program.	Mixed methods	PLWD (n = 20), care partner (n = 18)	Author developed questionnaires pre- and post-intervention analyzed with nonparametric statistics and thematic analysis of in-person or telephone interviews with sub-sample of participants.	Nonparametric statistics (Wilcoxon signed-rank test), thematic analysis

^a^Person living with dementia.

Of 10 studies that reported services, five delivered open-ended support (Carone et al., 2016; Clare et al., 2008; Davies-Quarrell et al., 2010; O’Connell et al., 2014; Phinney et al., 2016) while the remaining ran interventions for 6 months (Morhardt et al., 2019), 20 weeks (Marziali & Climans, 2009), 10 weeks (Jokel et al., 2017), 7 weeks (Diehl et al., 2003), and a single session (Taylor-Rubin et al., 2020). Duration (ranging from one to 6 hours) and frequency (weekly, bi-monthly, and monthly) also varied. Despite diverse delivery each program emphasized the value of peers coming together for listening, sharing, learning and/or social connection. In addition, recognition of a role for peers in supporting people with young onset dementia was reinforced by Stamou et al. (2020) in their survey of people living with dementia and care partners. A summary of peer support in each study is reported in Table 2.

**Table 2.** Description of peer support within an intervention (n = 11).

Author, date	Goals/Objectives	Conceptual background	Delivery	Outcomes
Carone, et al. (2016)	To explore the needs of people with early onset dementia and the impact of football and sports activities as a non-pharmacological intervention, on both the individual and their family members.	Football as a novel non-pharmacological intervention and drawing on the universality of the activity; literature suggesting benefits in other groups (schizophrenia and depression) and providing opportunities for “normalization of life” (p. 1372) to enhance mood and coping (De Boer et al., 2007); reference to Kitwood’s (1997) personhood and a “citizenship type model of community-based service provision” (p. 1317).	1.5-h weekly sports activities for men living with early onset dementia. Refreshment area for family members to sit and converse. Delivered by coaching staff in conjunction with local Alzheimer Society.	Benefits of peer support related to wives having the opportunity to interact and share problems with peers and gain respite. For PLWD^a^, reported benefits related more to engaging in physical activity providing opportunities to learn new skills, and meet new people, including other male PLWD.
Clare et al. (2008)	To promote respect and dignity for persons with dementia, provide a forum for information exchange, encourage support mechanisms, advocate for services and link people to support groups.	The social power of self-help groups (e.g., empowerment, shared values and norms, collective self-realization, advocacy and collective action) (Gray, 2001; Harvey et al., 2000; Reicher & Haslam, 2006).	Internet-based support network.	The network provided a forum for feeling understood, developing a sense of belonging, counteracting isolation and loneliness, giving and receiving support, gaining knowledge about dementia, and finding a voice to address stigma.
Davies-Quarrell, et al. (2010)	A peer support, relationship-centred club for younger people with dementia.	Relationship-centred care operationalized through the Senses Framework (Nolan et al., 2006).	Club members (PLWD, care partners and extended family, bereaved care partners) determine all activities and club staff provide a facilitative, educative, and supportive role.	Club described as a lifeline, providing companionship and support through difficult times.
Diehl et al. (2003)	1. To provide caregivers with a medical model of FTD, legal and financial advice, information on services. 2. To learn more about the needs of FTD care partners. 3. To encourage mutual support among care partners to stimulate coping strategies.	Differences in caring for someone living with AD vs someone living with FTD, primarily due to behavioural and personality challenges, necessitating need for diagnosis-specific support group.	1.5-h weekly physician moderated intervention for spousal care partners for 7 weeks comprising educational presentations and opportunities for interaction and followed by optional monthly self-help meetings.	Information about the disease, communicating with physicians and representing person’s interests rated positively and as contributing to lessening burden and helping with self-care. Positive rating of the opportunity to meet with peers and to share experiences.
Jokel et al. (2017)	1. To provide education on issues relevant to PPA. 2. To teach effective communication strategies. 3. To establish a safe forum for problem-solving, sharing experiences of PPA, practising strategies and learning from failures.	Communication and social participation are fundamental needs. Comprehensive functional interventions for people living with PPA are limited. Group approaches offer knowledge, problem-solving, coping skills and peer support.	2-h weekly session for 10 weeks comprising educational presentations, communication skills training and ‘safe forum’ for problem-solving and sharing led by speech language therapist. First hour was communication group with PLWD and separate education session for care partners followed by second hour practising communication strategies in dyads.	Reported outcomes included improved communication skills and knowledge of PPA including psychosocial issues. Some results were statistically significant. Participants highlighted that being understood by others in the group was important with spouses reporting that peer support was critical to success of intervention.
Marziali & Climans (2009)	To provide an internet-based program for spousal FTD care partners that is accessible regardless of location, available at time that are convenient to participants and replicates face-to-face information support groups. Session goals: 1. Encourage each group member to share their personal stories and experiences. 2. Help group members connect with one another. 3. Respond to the members’ anxieties about participating in the group. 4. Encourage group members to share problem-solving techniques.	Argues for tailored support for FTD spousal care partners because of stress, fatigue, anxiety, and impaired immunologic responses associated with social and psychiatric problems of the illness. Psychosocial interventions can delay nursing home admission.	1-h video-conferencing educational and support sessions for spousal care partners facilitated by a trained health care professional for 10 weeks followed by an additional10 weeks of mutual self-help.	Participants positively rated the intervention for its accessibility and the opportunity to connect with others in similar situations, share, be understood and receive emotional support around the daily challenges of caregiving.
Morhardt et al. (2019)	1. To describe the social, emotional, and educational needs of people living with PPA and their care partners. 2. To test the efficacy of a psycho-educational support program. 3. To explore the benefits and challenges of offering a psycho-educational support program.	Draws on ecological systems theory (Bronfenbrenner, 1979) and group practice model (Penninx et al*.*, 1999); also the value of psycho-education to increase resilience, coping skills and empowerment (Hayes & Gantt, 1992; Landsverk & Kane, 1998).	1.5-h bi-monthly sessions for 6 months, comprising 45 min for educational presentations and discussions by two trained facilitators; and 45 min for support groups, one for person living with PPA and one for care partners.	Report on outcomes for person living with PPA only: improved coping with language decline, expressing resilience through sharing compensatory strategies, sharing their diagnosis to confront stigma, improved self-confidence and a sense of group belonging.
O'Connell et al. (2014)	Support group for rural spouses of individuals with young onset dementia following a request for an emotion-processing group rather than psychoeducational group.	Emphasis on the concept of universality (Yalom, 2005); need for tailored support based on age of onset and diagnosis; virtual component was influenced by restrictions of travel burden for rural dwellers and value of videoconferencing telehealth for meeting support needs.	1.5-h open-ended monthly virtual meeting focused on emotion processing and facilitated by two clinical psychologists.	Opportunity for social connections among people in similar circumstances (diagnosis, age, relationship) was rated positively. Virtual format restricted extra-group conversations.
Phinney et al. (2016)	Independent social recreational group for people living with young onset dementia.	Importance of well-being and quality of life through physical, creative and leisure activities to provide pleasure, enable a sense of personal identity, wellbeing, and social connections. Also linked to social citizenship (Bartlett & O’Connor, 2010) described as opportunities for engagement in community life and freedom from discrimination.	Open ended social recreation day program operating 1–3 days per week for 6-h/day. Program run by 3 leaders and volunteers.	Activities provided a focus away from dementia, the club provided social connection and a sense of belonging, and a finding a place in their community.
Stamou et al. (2020)	To address challenges of diagnosis and lack of age-appropriate support for young onset dementia and provide evidence to inform age-appropriate service provision.	—	—	Themes reflected the importance of tailored support, around diagnosis and age. The accepting environment of peer support groups and the opportunity to share with others was rated positively.
Taylor-Rubin et al., 2020	To deliver a 3-h PPA-specific group education and support session for people living with PPA and care partners.	Need for support to address unique needs associated with diagnosis (progressive language impairment) distinct from support for memory-led or aphasia challenges.	Single 3-h intervention comprising 45 min disease education, 60 min psychoeducation and practical strategies, 45 min communication strategies and 30 min peer support. Delivered by speech language therapist and clinical psychologist.	Statistically significantly outcomes for care partners included knowledge, management of mood and opportunity to meet others.

^a^Person living with dementia.

### Quality assessment of evaluation methods

Of the 11 included studies, nine were qualitative studies, with the remaining being quasi-experimental and mixed methods research. When reported (n = 9), data evaluating the impact of the peer support were collected using interviews only (Clare et al., 2008; Marziali & Climans, 2009), interview and focus group (Carone et al., 2016), standardized questionnaire (Jokel et al., 2017), adapted and author developed questionnaire (Diehl et al., 2003), author developed questionnaire and interview (Taylor-Rubin et al., 2020), post group discussion, field notes and attendance records (O’Connell et al., 2014), interviews, focus group, observation, and satisfaction survey (Phinney et al., 2016), and field notes and group transcriptions (Morhardt et al., 2019). The paper that reported on post-diagnosis supports described an author developed qualitative questionnaire (Stamou et al., 2020).

There was a range of methodological quality in the studies included in the review. Within the qualitative studies, most papers (n = 7) neglected to define peer support, include a statement regarding the location of the researchers culturally or theoretically, and/or a statement commenting on the influence of the researcher on the research or vice versa (i.e., reflexivity). Four of these papers were also identified as lacking clarity in terms of how the conclusions were drawn from analysis. These issues reflect many of those also raised by Carter et al. (2020) and Dam et al. (2016). The remaining 4 papers were of good quality overall. The quality assessment of the papers is set out in Supplementary File 1. There was no evidence in the studies published after 2013 of the use of the Template for Intervention Description and Replication (TIDier) (BMJ, 2014) to enhance the quality of reporting on interventions.

### Theoretical and conceptual frameworks 

The next stage of analysis and synthesis led to the identification of three overarching peer support themes. These included: (1) peers as necessarily part of social support interventions; (2) a theoretical portmanteau; and (3) dementia spaces and relationality.

1. Peers as necessarily part of social support interventions

An examination of findings specific to the contribution of peers demonstrated that, without exception, peers were viewed as an essential element within support programs for people living with rare or young onset dementia. The inclusion of peers as a necessary ingredient within multi-component support was also reinforced in Stamou et al.’s (2020) survey results. No authors identified any negative results emerging from peer involvement, although O’Connell et al. (2014) expressed concern about the management of extra-group relationships.

The positioning of peers to achieve support outcomes within a community-based service (e.g., Carone et al., 2016; Davies-Quarrell et al., 2010; Phinney et al., 2016) appeared to be distinctly different than that in outpatient interventions (e.g., Jokel et al., 2017; Morhardt et al., 2019; Taylor-Rubin et al., 2020). For community-based services the staff role was primarily facilitative and the emphasis on social participation and support through peers appeared to occur more naturally through walking (Phinney et al., 2016) or football (Carone et al., 2016), for example.

Within the outpatient interventions it was difficult to establish the exact nature of the support provided by peers, apart from peer discussions, due to detailed description being largely absent. Outpatient interventions were professionally designed apart from O’Connell et al. (2014) who involved service users in the design of a “emotional-processing group” (p. 386). In contrast to community programs, these were time-limited and most often characterized by diagnosis specific psychoeducation with scheduled peer support time (Jokel et al., 2017; Morhardt et al., 2019; Taylor-Rubin et al., 2020), or optional self-help following the professionally led education (Diehl et al., 2003). Here too, the psychologist’s, speech language therapist’s, physician’s or social worker’s role was in the foreground. The interventions targeted both people living with dementia and care partners, in the instance of PPA, and only care partners for people living with FTD. In addition, there were more flexible group agendas that purposively focused on virtual peer sharing among care partners despite being professionally facilitated (Marziali & Climans, 2009; O’Connell et al., 2014). Marziali and Climans (2009) also offered a 10-weeks self-help group at the conclusion of the formal sessions. It is worth noting here that the outcomes for the groups with a psychoeducation component, and evaluated using pre/post questionnaires, appeared to demonstrate participants valued the professional contribution followed by peer engagement. This was similarly reported by Stamou et al. (2020) where opportunities for social participation including “camaraderie” and “sharing with others” (p. 5) followed specialist advice and information, age-appropriate services, and interventions for physical and mental health.

To what extent do support group members need to share similar characteristics and how does this impact on outcomes? The most homogenous support was offered by O’Connell et al. (2014) with care partner participants purposefully sharing similar age, relationship, and partner’s diagnosis (8 of 10 participants caring for someone living with FTD). It was not clear, however, if the extent of homogeneity here had any significant impact on outcomes. Community-based supports focused on younger age, and Clare et al.’s (2008) on-line self-help group and Davies-Quarrell et al.’s (2010) club model were also characterized by stage of dementia (early to mid). Outpatient interventions were condition specific (i.e., FTD, PPA) and emphasized the need for tailored psychoeducational support as opposed to the more commonly offered generic dementia education. Where care partners were involved most were female and spousal, although this appeared to be by chance (Carone et al., 2016; Marziali & Climans, 2009; Morhardt et al., 2019; Taylor-Rubin et al., 2020). Peer support for male care partners was notably absent. Stamou et al.’s (2020) survey to document post-diagnostic support needs identified the demand for age versus diagnosis tailored support.

2. A theoretical portmanteau

The theoretical foundations for each study, how they conceptualized both peer support and the lives of people affected by rare or young onset dementia, were both explicit and implicit but providing some evidence of the authors’ conceptual alignment. A blend of conceptualizations or ideas which we identified as a theoretical portmanteau were more common yet sometimes difficult to attribute to peer support specifically.

Peer support emerging from community-based programs seemed to be more affiliated with understandings emerging from self-help, personhood or person-centred care, the social model of disability and social citizenship. Clare et al. (2008) were unique in their exploration of digital self-help for people living with dementia arguing that both face-to-face meetings and professional involvement were not necessary to achieve coping benefits from support from peers. The authors also reported that self-help permitted the development of collective social identities which would in turn create social and political power to enable advocacy and change efforts (Gray, 2001; Harvey et al., 2000; Reicher & Haslam, 2006). The “accepting social environment of peer support” and opportunities to “have a voice” or “raising awareness on young onset dementia” (p. 6) were also identified by Stamou et al. (2020). The use of self-help in outpatient support for care partners was not expanded on or evaluated (Diehl et al., 2003; Marziali & Climans, 2009).

Explanations using or inferring Kitwood’s personhood or personalizing cultures (1997), normalization and social role valorization (Thomas & Milligan, 2018), and the right to full citizenship participation with and among peers for people living with dementia (Bartlett & O’Connor, 2010) were features in Davis-Quarrell et al. (2010) and Phinney et al. (2016) and implied in Carone et al. (2016). As Phinney et al. (2016) stated “…citizenship is not a fixed status but is performed through everyday experiences of movement and mobility…guided by a philosophy that foregrounds the importance of continued participation in activities they consider to be normal” (p. 389). This deviation from a deficit model to a strengths-based one, enablement and a relationship-based approach was underscored by Davis-Quarrell et al. (2010) and their use of the Senses Framework to examine the outcomes a club model for people with young onset dementia (Ryan et al., 2008). Remarkably, the Senses Framework dismissed a hierarchy among peer groups in their club model (i.e., person living with dementia or care partners or staff) and argued that relationship-centred care was achieved when all senses (achievement, belonging, continuity, purpose, security, and significance) were experienced among all groups, including staff (Ryan et al., 2008).

Elements of the mental health recovery model, including self-help, biomedical conceptualizations of caregiving stress and burden, Yalom’s (2005) therapeutic factors for group psychotherapy, and ecological systems theory were evident in the outpatient peer support interventions. Recovery model concepts such as connection with others, empowerment, meaning and identity (Leamy et al., 2011) and recovery capital (Tew, 2013) were evident to some degree in Diehl et al. (2003), Jokel et al. (2017), Marziali and Climans (2009), Morhardt et al. (2019), O’Connell et al. (2014) and Taylor-Rubin et al., 2020. Although overlapping with personhood conceptualizations, the emphasis in these relational support interventions for both people living with dementia and care partners appeared more aligned with the development of recovery capital and centering personal efficacy as well as longer-term coping.

The biomedical binary of coping dementia care partner/not coping dementia care partner was also evident in Diehl et al. (2003), Jokel et al. (2017) and Marziali and Climans (2009). Interventions that were delivered by psychologists (O’Connell et al., 2014; Taylor-Rubin et al., 2020) placed considerable importance on Yalom’s (2005) universality and altruism factors, although delivering support groups as opposed to group psychotherapy. Finally, Morhardt et al. (2019) introduced ecological systems theory (Bronfenbrenner, 1979) to explain how people “understand and cope with their illness in relation to others” (p. 1312), and group psychoeducation as a means of expanding social networks alongside normalizing experiences, developing tools for self-care (Penninx et al., 1999) and coping and empowerment (Hayes & Gantt, 1992; Landsverk & Kane, 1998). These conceptualizations were not returned to in their discussion of outcomes, and where relevant, if these were more salient for people living with dementia and/or care partners.

3. Dementia spaces and relationality

Whereas an evidence-informed model of peer support did not emerge due to the varied population and nature of the evaluations conducted, the studies encouraged valuable reflection on the grounds that individuals affected by dementia inhabit previously unimagined social spaces taking on meaning in relation to others who inhabit similar spaces. Moreover, there was a recognition that a sense of belonging via peer support (i.e., inclusion) was a requested space for people affected by rare or young onset dementia (e.g., Carone et al., 2016; Clare et al., 2008; O’Connell et al., 2014; Stamou et al., 2020). Yet peer support was a scarce resource in the broader community of dementia care services (e.g., Davis-Quarrell et al., 2010; Jokel et al., 2017; Marziali & Climans, 2009; Taylor-Rubin et al., 2020). Importantly, these were relational spaces where all individuals expressed, practiced, and shared their new identities.

These studies recognized, largely using qualitative data, a variety of relational features common within coping networks or achieved during opportunities for social participation. These features remained more or less visible in formal multi-component groups, informal social/recreational programs or a self-help network, and whether they were delivered virtually or face-to-face. Relationality among others who were similar, whether a person living with dementia or a care partner, was reported as feeling normal and described as inclusive (Carone et al., 2016; Clare et al., 2008; Davis-Quarrell et al., 2010; Diehl et al., 2003; Marziali & Climans, 2009; Morhardt et al., 2019; O’Connell et al., 2014; Phinney et al., 2016; Taylor-Rubin et al., 2020). The relational aspects of these peer environments for care partners were also portrayed in terms of group reciprocity. The reciprocal nature of the groups through the sharing of experiential knowledge was thought to have positively promoted interpersonal competence and personal affirmation (Clare et al., 2008; O’Connell et al., 2014; Taylor-Rubin et al., 2020). Both Clare et al. (2008) and O’Connell et al. (2014) described reciprocity as reinforcing shared values and motivating genuine advocacy efforts for awareness raising and/or enhanced services. An engagement in advocacy activities to create positive social change may be an important feature within peer support for people who are younger in age.

Given dementia spaces also meant navigating change due to, for example, neurodegenerative decline or care transitions, relational safety among peers also seemed to be highly valued among study participants (Carone et al., 2016; Davis-Quarrell et al., 2010; Jokel et al., 2017; O’Connell et al., 2014). For example, Davis-Quarrell et al. (2010) using the Senses Framework (Ryan et al., 2008) described a sense of security as permission to be vulnerable in a supportive environment which in turn fostered personal growth. Jokel et al. (2017) described the value of people living with PPA practicing communication strategies with uncritical peers. Whereas community-based studies where focus was on the person living with young onset dementia there also appeared to be an emphasis on relational autonomy (Perkins et al., 2012). The creation of inclusive spaces and opportunities for social participation with peers supported both a participant’s selfhood and capabilities which, in turn, maintained both agency and autonomous action (Carone et al., 2016; Clare et al., 2008; Davis-Quarrell et al., 2010; Stamou, et al., 2020).

## Discussion

The purpose of this literature review was to examine the characteristics of peer support for people affected by rare or young onset dementia, including benefits of participation and how this had been evaluated. Consistent with a much larger body of work examining peer involvement in social interventions, this review reinforced the valuable contribution of peers despite a full understanding of mechanisms of change not achieved. Notwithstanding, enthusiasm for peer support will likely remain, if not expand, and we thus draw attention to various considerations as these forms of support continue to be conceptualized, delivered, and evaluated.

### Theorizing peer support

Thinking around who, why and how peers are included in support interventions for people affected by dementia continues to escape thorough theoretical clarity – that is, theorizing both the peer support and the characteristics and needs of the peer population. This is not unsurprising given that our theorizing about dementia, the complex lives of those affected, and policy and practice responses to the needs of people living with dementia are evolving across different disciplines. Many theoretical approaches have been criticized for either under theorizing structural influences on the lives of people living with dementia or avoiding how various constructs are applied at a practice level. In both instances these approaches neglect those in later the stages of their illness (Milne, 2020) and those who are living with an atypical or young onset dementia (Brotherhood et al., 2019). Studies in this review have not escaped these challenges.

Most studies in this review favoured an eclectic approach to peer support, yet congruent with explanations featuring elements from the recovery model, including self-help, disability studies and person-centred care. In other studies, these ideas informed the why but were not always articulated in terms of the who and how of peer involvement. For example, Phinney et al. (2016) argued the relevance of social citizenship to inform social participation with peers for people living with young onset dementia. While recognizing human rights and agency are valuable constructs for people living with dementia who are often denied these, clarity regarding how these informed the delivery of support were limited. Davis-Quarrell et al. (2010) adopted the lesser-known Senses Framework in their club model. The Framework positively focused on relationships among peers and others, but the study’s methods and analysis provided limited clarity on how the Framework guided any peer support delivery to the target population.

Arguably, the need for a broader and critical conceptual lens in dementia care delivery has been long recognized (Higgs & Gilleard, 2016), and yet any new or emerging conceptualizations were not explored within these studies. The exception was Davis-Quarrell et al. (2010), however, the study design made it difficult to extrapolate ideas for replication elsewhere. The complexities in peer support or peer influence for younger people with an atypical diagnosis due to everchanging family roles, neurodegenerative decline, and psychosocial transitions specific to age and stage were left largely underdeveloped. Despite the larger number of female care partners, normative assumptions around dementia caregiving were left unchallenged within peer delivery. Constructs around emotional labour, female identities, gender differentiated help seeking behaviours and others (Erol et al., 2016; Gilhooly et al, 2016; Pöysti et al., 2012), were only inferred by Marziali and Climans (2009) and O’Connell et al. (2014), and neglected elsewhere (e.g., Carone et al., 2016). Consumer driven or co-produced peer support models which complement both social disability and social citizenship paradigms, were not apparent among those studies aligned here although evident in Clare et al.’s (2008) report on self-help and O’Connell et al.’s (2014) emotional-processing group. Further, the desire to engage in advocacy efforts as identified by Clare et al. (2008) and O’Connell et al. (2014) required further explanation in terms of the target population and how this could be realized at a practice level. And finally, newer constructions emerging from relationship-centred or relational citizenship models (Kontos et al., 2017) were not explicitly expanded on, although resonating within some of the study findings and how these were discussed.

### Characterizing the delivery and evaluation of peer support

Both community-based non-profit organizations and out-patient health services were responsible for the provision of peer support interventions asserting the need for tailored support given age and/or diagnosis. This rationale is supported elsewhere (e.g., Queluz et al., 2020). The role of peer support within a continuum of care and how this interacted with other health or social care services for people living with rare and young onset delivery was not illustrated in any study. Their delivery varied in a number of important ways that was no doubt a reflection of both funding and the extent of professional involvement. Community-based involvement in peer support was characterized by social participation and relationship development, particularly for people living with dementia who were younger in age. As Phinney et al. (2016) noted, dementia was not the focal point within these social networks. By and large outpatient peer support delivery for people living with dementia and care partners was time-limited, included with psychoeducation and its emphasis on dementia knowledge and coping skills. Three studies reported on virtual delivery with positive outcomes similar to face-to-face groups. This finding also echoes Carter et al. (2020) in their scoping review of peer interventions for dementia care partners. This is particularly timely given our current context, and recent shifts in thinking about the potential for virtual delivery in terms of reaching a population that is geographically dispersed or accommodating care partners and their caring or employment responsibilities.

Discerning the contribution of peer-led versus professional-led peer support, the ideal extent of heterogeneity or homogeneity among peers, and support for people living with dementia versus care partners was difficult to establish. Visible gaps in our understandings included peer support for children, parents or siblings who also play important caring roles and yet understudied and unnoticed in support programs (Roach et al., 2016), the role of peers for individuals where their dementia is in the later stages and/or their care partners, and ethnocultural, linguistic and other diverse groups where both the life course and inequalities require critical attention (Milne, 2020).

Regarding intervention evaluation, the studies reporting on peer support delivery focused on in-depth service descriptions including feedback from users (Carone et al., 2016; Clare et al., 2008; Davies-Quarrell et al., 2010; Marziali & Climans, 2009; Morhardt et al., 2019; O’Connell et al., 2014; Phinney et al., 2016) and outcome evaluations with an emphasis on the professional component of delivery (Diehl et al., 2003; Jokel et al., 2017; Taylor-Rubin et al., 2020). The contribution from the qualitative studies was welcomed given the complexities in support delivery. Though their lack of conceptual development and methodological rigour was at times disappointing, these contributions were important to stimulate further theorizing and reinforcing the need to develop more rigorous evaluation designs. In the case of outcome evaluations, the absence of a theory of change or logic model, where relevant, may have impeded the development of a more effective evaluation strategy including both process and outcome evaluations. An emphasis on the latter meant that the how (e.g., resources, activities, decisions) specific to delivery were not set out, reported on, or analyzed. The absence of process evaluations of peer interventions to inform randomized clinical trials was also recently identified by Walker and Peterson (2021).

### Strengths and limitations

The strength of this literature review was that it provided an integrative synthesis of peer support for people affected by rare and young onset dementia which is an under-researched population. The review and synthesis also followed established protocols including an evaluation of the quality of the research, although challenging at times given differing methodological and analytical approaches. Our synthesis of how peer interventions were theorized adds an important contribution given this is often unaddressed in other reviews. The review is limited, however, due to the small number of papers that met the inclusion criteria. A review of studies addressing other rare conditions or chronic long-term illness in younger populations may have provided us with other valuable insights. The results of our quality review where authors neglected to address both theoretical and researcher bias or other views affecting reported outcomes also impacted on our own conclusions.

## Conclusion

The lack of recognition of dementia diversity within the largely homogenous dementia care sector has resulted in people affected by rare and young onset dementia being denied opportunities to participate in tailored peer support. A growing body of literature on living with an atypical condition is now casting a light on varied dementia spaces characterized by, among others, health, illness, loss, change and caring, and in doing so, acknowledging the possibilities for peer support models purposefully directed to reach those previously forgotten. The broader issue of studies neglecting to sufficiently conceptualize and describe interventions is an important one – drawing attention to the need to continue to explore varied and innovative delivery (e.g., co-produced models) and robust process and outcomes evaluation methods to inform support delivery within the dementia care sector.

This work is part of a larger study exploring tailored and continuous multi-component support for people affected by rare or young onset dementia, including the contribution of peers. The important insights gathered here will contribute to the further exploration of models of support provision and their evaluation in the practice sector.

## Supplemental Material

Supplemental Material - Peer support for people living with rare or young onset dementia: An integrative review Click here for additional data file. Supplemental Material for Peer support for people living with rare or young onset dementia: An integrative review by Mary Pat Sullivan, Veronika Williams, Adetola Grillo, Roberta McKee-Jackson, Paul M Camic, Gill Windle, Joshua Stott, Emily Brotherhood and Sebastian J Crutch in Dementia
