# Supplementary material for: Peer support for people living with rare or young onset dementia: An integrative review
Source: Dementia (London). 2022 Sep 16;21(8):2700–26. doi: 10.1177/14713012221126368 (PMC9583292; doi:10.1177/14713012221126368)
Supplement: Supplemental Material - Peer support for people living with rare or young onset dementia: An integrative review [file sj-pdf-1-dem-10.1177_14713012221126368.pdf]

Supplementary File 1

Quality Assessment of Qualitative Studies (n=9)

JBI Checklist Tool (2020)	Carone, et al (2016)	Clare et al. (2008)	Davies-Quarrell et al. (2010)	Diehl et al. (2003)	Marziali & Climans (2009)	Morhardt et al. (2019)	O'Connell et al. (2014)	Phinney et al. (2016)	Stamou et al. (2020)
Congruity between stated philosophical perspective and research methodology	?	+	?	-	-	+	-	+	?
Congruity between research methodology and research question or objectives	?	+	?	?	-	+	-	+	+
Congruity between research methodology and data collection methods	+	+	?	?	-	+	?	+	?
Congruity between research methodology and representation and analysis of data	+	+	?	?	-	+	?	+	+
Congruity between research methodology and interpretation of results	+	+	?	-	-	+	?	+	+
Statement locating researcher culturally or theoretically	-	-	+	-	-	?	-	-	-
Influence of									

researcher on research, and vice- versa, addressed	-	-	-	-	-	-	-	?	-
Participants, and their voices, adequately represented	+	+	+	?	-	+	+	+	+
Research ethical according to current criteria or evidence of ethical approval by an appropriate body	+	+	-	-	-	+	_	+	+
Conclusions drawn flow from analysis or data interpretation	+	+	-	-	?	+	?	+	+
(+) = yes; (-) = no; (?) = unclear/can't tell									

Quality Assessment of Quasi-Experimental and Mixed Methods Studies (n=2)

JBI Checklist Tool (2020)	Jokel et al. (2017)	Mixed Method Appraisal Tool (2018)	Taylor-Rubin et al. (2019)
Clarity on 'cause' and 'effect'	+	Clear research questions	+
Participants included in any comparisons similar	+	Data addresses research questions	+
Participants included in any comparisons receiving similar treatment/care, other than the exposure or intervention of interest	?	Is the qualitative approach appropriate to answer the research question?	+
Control group	+	Are the qualitative data collection methods adequate to address the research question?	+
Multiple measurements of outcome both pre and post intervention	+	Are the findings adequately derived from the data?	+
Follow up complete or group differences adequately described and analyzed	?	Is the interpretation of results sufficiently substantiated by data?	-
Outcomes of participants included in any comparisons measured in the same way	+	Is there coherence between qualitative data sources, collection, analysis and interpretation?	-

Outcomes measured in a reliable way	+	Is the sampling strategy relevant to address the research question?	+
Appropriate statistical analysis used	+	Is the sample representative of the target population?	?
		Are the measurements appropriate?	?
		Is the risk of nonresponse bias low?	?
		Is the statistical analysis appropriate to answer the research question?	+
(+) = yes; (-) = no; (?) = unclear/can’t tell			
